# Exploring the Efficacy of Novel Therapeutic Strategies for Periodontitis: A Literature Review

**DOI:** 10.3390/life14040468

**Published:** 2024-04-03

**Authors:** Casandra-Maria Radu, Carmen Corina Radu, Emil-Marian Arbănaşi, Timur Hogea, Viorela Romina Murvai, Ioana-Andreea Chiș, Dana Carmen Zaha

**Affiliations:** 1 Doctoral School of Biological and Biomedical Sciences, University of Oradea, 1 University Street, 410087 Oradea, Romania; 2 Department of Forensic Medicine, George Emil Palade University of Medicine, Pharmacy, Science and Technology of Targu Mures, 38 Gheorghe Marinescu Street, 540139 Targu Mures, Romania; 3 Institute of Forensic Medicine, 540141 Targu Mures, Romania; 4 Doctoral School of Medicine and Pharmacy, George Emil Palade University of Medicine, Pharmacy, Sciences and Technology of Targu Mures, 540142 Targu Mures, Romania; 5 Clinic of Vascular Surgery, Mureș County Emergency Hospital, 540136 Targu Mures, Romania; 6 Department of Vascular Surgery, George Emil Palade University of Medicine, Pharmacy, Science and Technology of Targu Mures, 38 Gheorghe Marinescu Street, 540139 Targu Mures, Romania; 7 Department of Preclinical Disciplines, Faculty of Medicine and Pharmacy, University of Oradea, 1 December Sq, 410028 Oradea, Romania; 8 Department of Oral Rehabilitation, Faculty of Dentistry, University of Medicine and Pharmacy “Iuliu Hatieganu”, 400012 Cluj-Napoca, Romania

**Keywords:** periodontitis, essential oils, novel therapies, oral microbiome

## Abstract

Periodontitis, a prevalent oral condition, is facing difficulties in therapeutic approaches, sometimes leading to failure. This literature review was conducted to investigate the diversity of other therapeutic approaches and their potential contributions to the successful management of the disease. This research scrutinized the alterations in microbial diversity and imbalances in crucial microbial species, which contribute significantly to the pathogenesis of periodontitis. Within the limitations of this study, we highlight the importance of understanding the treatment plan’s role in periodontitis disease, opening the way for further research and innovative treatment plans to mitigate the impact of periodontitis on oral health. This will aid both healthcare professionals and patients in preventing and effectively treating periodontitis, ultimately improving oral health outcomes and overall systemic health and well-being.

## 1. Introduction

Periodontitis is a condition characterized by the gradual deterioration of tooth-supporting tissues (root cementum, periodontal ligament, alveolar bone, and gingiva), and can lead to tooth loss [1,2].

Over the years, various classifications of periodontal disease have been proposed to better delineate and categorize the diverse pathologies encompassed within this spectrum. These classifications serve as valuable tools for research, therapeutic interventions, and epidemiological studies [3]. 

It can be seen in up to 30% of the global population, and together with dental caries, it is one of the most common oral diseases and is affected not only by local factors, but also systemic etiological ones [4,5,6,7]. Clinical symptoms include loss of attachment, radiographically evident bone resorption (vertically and horizontally), the appearance of periodontal pockets, and palpable bleeding. This loss is often accompanied by the formation of periodontal pockets because they provide a favorable environment for the accumulation of harmful bacteria [8,9]. It also involves complicated dynamic interactions between specific bacterial infections, damaging host immune responses, and environmental factors such as smoking [10,11]. Aside from its significance as a dental ailment, periodontitis has grown in prominence as it unequivocally evolves into a systemic syndrome characterized by unresolved hyperinflammation, disturbance of the immune system, and other clinical symptoms such as changes in color, contours, and consistency and bleeding on probing [6,12,13,14,15]. 

Periodontitis is exceedingly difficult to treat due to the disease’s etiology, complications that arise after anti-microbial therapy, and a lack of knowledge about the interactions that occur between different types of bacteria during the disease [16,17]. Dental biofilm is an etiological factor of periodontal diseases; when attached to teeth, it consists of polymers and is resistant to host defenses [16,18,19]. This matrix is composed of extracellular polysaccharides and glycoproteins, providing a protective environment for microbes in the dental biofilm [20,21]. 

Therapeutic approaches targeting restoring a balanced oral microbiome with antimicrobial therapy, probiotics, and personalized interventions (laser, photodynamic methods, ultrasonic scaling, and scaffolding interventions) are being explored to manage, and also prevent, the development and progression of periodontitis [22,23]. Over many years, this disease normally manifests few or only minor symptoms, which are frequently not perceived or classified by the patient [24,25]. Even if the treatment of this disease is simple and cost-effective if diagnosed in the early stages, it causes losses of billions of dollars globally each year [26,27]. Thus, early periodontal diagnosis is a critical strategy for facilitating quicker and more effective therapies by achieving a better long-term prognosis [17,28]. Also, some patients lose their teeth due to chronic periodontitis, which leads to mental health issues and depression [29].

Emphasizing routine dental check-ups, good oral hygiene practices, and the use of natural oral care products can maintain a healthy oral microbiome and prevent periodontitis. While chlorhexidine remains the benchmark treatment for periodontitis and is widely regarded as the gold standard treatment due to its potent antimicrobial properties and established efficacy in controlling plaque accumulation and reducing gingival inflammation, emerging therapies are showing promising potential [30,31]. By unravelling the intricate relationship between systemic health and periodontitis, researchers and healthcare professionals can work toward more effective strategies for oral disease prevention and management.

## 2. Main Body

### 2.1. Materials and Methods 

Objectives: 

Through an extensive review of the existing literature, we intended to analyze the current understanding of other therapeutic possibilities by discussing their advantages, disadvantages, therapeutic properties, and administration methods.

Study selection: 

Inclusion criteria considered randomized clinical trials published in the last 10 years that evaluated the results of novel therapeutic approaches for periodontal diseases. The exclusion criteria included patients who had systemic diseases that could influence the progression of periodontitis, pregnant women, underaged children (<18 years old), and smokers. 

Data extraction:

An extensive electronic literature search was conducted on the following databases: PubMed, PMC, ScienceDirect, and Scopus using Medical Subject Heading (MeSH) terms: biofilm, oral microbiome, novel therapies, essential oils, oral care products, and periodontitis. The search was performed from January 2023 to October 2023. A reviewer (C.-M.R.) screened the titles for eligible articles. If the articles did not present any eligibility criteria in the title, the article was read in detail. Not all relevant publications were listed in the selected databases, and not all relevant publications were written in English. We selected randomized clinical trials assessing the effects of using novel therapy approaches for periodontitis published in the last 10 years and written in English. The titles and abstracts of the papers were downloaded through the Mendeley application and categorized as suitable or non-suitable, and duplicates were deleted.

### 2.2. Periodontitis and Its Pathogenesis

**Figure 1:**
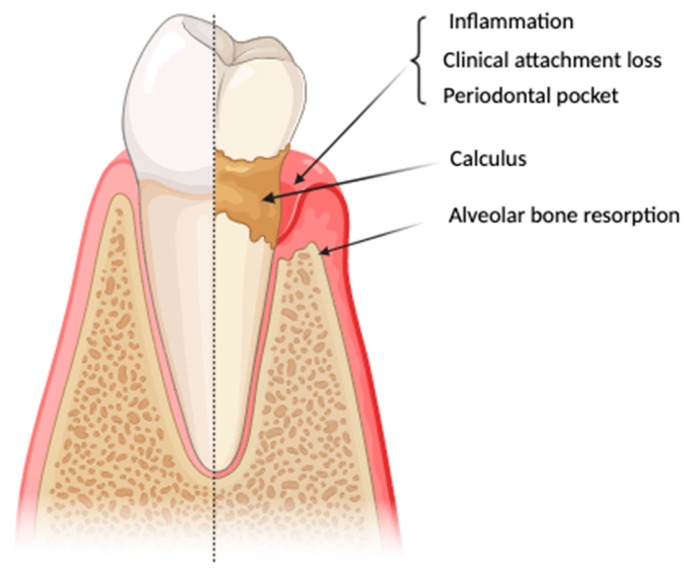
Key points for diagnosing periodontitis (created at www.BioRender.com (accessed on 2 October 2023)).

Periodontitis is a chronic, multifaceted inflammatory disease that represents a public health problem because it is predicted to increase in the future [17]. It is manifesting, as seen in Figure 1, gingival inflammation, clinical attachment loss, the radiographic-assessed resorption of alveolar bone, the development of periodontal pockets, gingival bleeding on probing, teeth mobility, and, in advanced stages, premature teeth loss and depression [32,33,34,35].

Periodontitis results from a disruption of the oral microbiome balance, as these diverse communities usually coexist in equilibrium. This imbalance, called dysbiosis, is often marked by an overgrowth of pathogenic bacteria and a decline in beneficial species. The shift toward a more pathogenic microbiota triggers an inflammatory response in the gums and periodontal tissues, leading to damage and bone loss [36,37]. The most pathogenic species are *Porphyromonas gingivalis*, *Tannerella forsythia*, and *Treponema denticola*, which contribute to the formation of biofilms and subsequent tissue destruction. They also produce toxins and enzymes that cause inflammation and tissue breakdown [38,39]. *Porphyromonas gingivalis* weakens host defenses in ways that promote the growth and development of the entire microbial population. Its potential to change the nutritional base for the microbial community causes considerable modifications to the community’s makeup [40].

These characteristics distinguish it as a keystone pathogen, a microbe capable of altering the ratios of other microorganisms within an ecological niche. The resulting disturbance of the proportionate relationship between symbionts and pathobionts activates the destructive cascade, resulting in inflammation and bone loss [41,42]. *Enterococcus faecalis*, while primarily associated with infections outside the oral cavity, has increasingly been implicated in periodontal diseases; it can exacerbate periodontal inflammation by inducing host immune responses characterized by the release of pro-inflammatory cytokines and chemokines and modulating the microbial composition of the periodontal pocket by favoring the growth of pathogenic species associated with periodontal disease [43,44,45].

### 2.3. Treatment Plan

The treatment must begin early, and it is focused on removing the key etiological component, the subgingival bacterial biofilm; can be surgical or non-surgical; and aims at controlling infection, reducing inflammation, arresting disease progression, and restoring periodontal health [6,14,15,46]. First, a clinical examination must occur. It involves the estimation of local factors, the development of discrepancies in teeth leading to increased plaque accumulation (for example, narrow interdental spaces), bleeding on probing, periodontal pocket depth estimation, the determination of recession, clinical attachment level, and furcation involvement [47,48]. Radiographic examination is also mandatory [37,49]. Non-surgical therapy is often the initial treatment plan, and it involves root planning and scaling to remove dental plaque and biofilm. This step will promote healing and reduce inflammation. Therefore, the main role of periodontal therapy is to improve gingival health and oral microbiome health, as well as preserve the remaining periodontal tissue [14,34]. 

Periodontitis is extremely difficult to treat due to the nature of the disease, the complications arising after anti-microbial therapy, and the lack of information regarding the interactions that occur between the different types of bacteria during the disease [50]. Non-surgical therapy involves mechanical methods of plaque removal, such as brushing, flossing, and scaling, which may lead to an incomplete removal of interdental bacteria [9,51]. The mechanical methods mentioned above are insufficient for removing interdental bacterial plaque, and in the absence of effective oral hygiene, initial dental plaque formation on a clean tooth occurs within 48 h [52,53,54]. 

Although mechanical periodontal treatment has shown great success, only when a probing depth smaller than or equal to 5 mm is reached once the tissue and bone begin their loss development, this loss is permanent, which is why a complementary weapon is more than welcome [55,56]. It is difficult to achieve healthy soft tissues due to antibiotic-resistant bacterial biofilm, and after its removal, there can rarely be tissue regeneration, as the severity of this disease depends not only on the bacterial load, but also on the host’s immune response [16,57,58].

The local antibiotic treatment scheme comprises minocycline microspheres and doxycycline, and systemic treatment involves a regimen of amoxicillin, metronidazole, azithromycin, and doxycycline, with dosages depending on the severity of the disease and other general aspects of the patient [9,48,59,60,61,62,63]. The systemic antibiotic treatment scheme involves different combinations, such as amoxicillin and metronidazole, to combat periodontal pathogens, but they may not be effective on all strains, especially *P. gingivalis* [38]. Plastic surgical therapy is necessary when non-surgical therapy is ineffective and includes soft-tissue grafting to cover exposed root surfaces and bone grafting to provide support for future implants [64,65,66,67]. It removes necrotic tissue and regenerates lost periodontal structures. Surgical procedures include flap surgery, bone grafting, guided tissue regeneration, and gingival grafting. Biomaterials used in surgical treatments provide better tissue regeneration, and researchers are developing them to copy the benefits of human tissues [68,69]. 

The periodic monitoring and evaluation of the periodontal status are essential for assessing treatment outcomes, monitoring disease progression, and changing the treatment plan, when necessary. These include regular dental check-ups, periodontal examinations, radiographic assessments, and comparing clinical parameters such as pocket depth and attachment levels [70].

### 2.4. Novel Therapies

Novel therapies for periodontitis represent a frontier in oral healthcare, aiming to address the complex pathogenesis of periodontitis and enhance treatment outcomes. These innovative approaches encompass a diverse range of strategies targeting microbial dysbiosis, inflammation, tissue regeneration, and host immune responses [71,72,73]. 

Essential oils, also referred to as “volatile oils”, are secondary metabolites produced by aromatic plants and are notable for their potent aroma. Derived primarily from various plant parts like leaves, fruits, resins, seeds, woods, barks, and berries, they capture the essence of the plant, including its taste and odor. EOs have garnered attention from research groups due to their potential in developing novel solutions for improving oral hygiene and also periodontitis treatments [74,75]. 

As stated by the World Health Organization, 80% of the world’s population relies on conventional medicine (herbal) for primary care. In nations with limited resources, herbs, and their derivatives account for 25% of all medical treatments utilized. China and India are two of the world’s most populous countries, and herbal medicine has been used for over 2000 years to treat mouth illnesses, including periodontal disease [76]. A new trend is growing in the use of herbal medicine as both a nutritional supplement and a dental therapy. 

Herbal compounds with antibacterial characteristics are used in toothpastes, mouthwashes, and other dental care products to reduce bacterial adhesion and plaque formation [77]. 

Researchers are exploring EOs to make them promising candidates for combating oral pathogens and reducing inflammation associated with periodontal diseases, as seen in Table 1. Studies have demonstrated the efficacy of essential oils, either alone or in combination with conventional therapies, in reducing plaque accumulation, gingival inflammation, and pocket depths in patients with periodontitis [78]. Furthermore, the use of EOs in mouthwashes, gels, or as adjuncts to scaling and root planning procedures has shown promising results in improving clinical outcomes and patient satisfaction, as seen in Tables 2 and 3.

One promising avenue of research involves the development of targeted antimicrobial agents that selectively eliminate periodontal pathogens while preserving beneficial oral microflora. These agents may include antimicrobial peptides, nanoparticles, and photodynamic therapy, which offer the potential for enhanced efficacy and reduced side effects compared to traditional antimicrobial agents [66]. Peptides of human origin show great potential as therapeutic agents with low host toxicity. Furthermore, the coevolution of these peptides with the oral microbiota shows that they may result in decreased levels of bacterial resistance [79]. Nanoparticles have acquired popularity in an array of uses in dentistry due to their distinctive characteristics, which make them suitable for drug administration. Their small size allows them to deliver drugs to specific tissues, cells, or pathogens in periodontal pockets. Periodontitis can be treated with a variety of nanomaterials, such as liposomes, lipid and polymeric nanoparticles, and dendrimers. Cell delivery systems have also been developed, including nano-capsules, nano-scaffolds, nano-coatings, and nano-shells [80]. An in vivo study concluded that using photodynamic therapy combined with traditional mechanical therapy resulted in considerably larger reductions in gingival bleeding on probing and gingival inflammation, which may result from the reduction in pro-inflammatory mediators [81].

Another emerging area of interest is immunomodulatory therapy, which seeks to modulate the host immune response to better control inflammation and tissue destruction in periodontitis. Leukocyte and inflammatory molecule activities increase and can lead to reducing inflammation, but these reactions can severely damage the alveolar bone and periodontal tissue. Modifying the immune microenvironment is a viable method for treating periodontitis, for reaching better levels of immune control and tissue repair, and for realizing innovative anti-inflammatory and periodontal regeneration therapy [70]. This approach involves the use of biologics, such as monoclonal antibodies and cytokine inhibitors, to target specific immune pathways involved in periodontal pathogenesis. The use of vaccinations against key pathogens aims to directly regulate the host immune responses. Furthermore, regenerative therapies aim to promote the regeneration of periodontal tissues damaged by disease, including bone, cementum, and periodontal ligaments. These therapies may involve the use of growth factors, stem cells, tissue engineering techniques, and scaffolds to stimulate tissue repair and regeneration in periodontal defects [82].

Additionally, adjunctive therapies, such as probiotics, prebiotics, and host modulation agents, are being investigated for their potential to enhance the effectiveness of conventional periodontal treatment modalities, such as scaling and root planning. Pre- and probiotics can come in different forms, such as liquid, paste, and solid. They act as an anti-inflammatory and antibacterial agent and can adhere to oral tissue by colonizing the epithelial cells and altering the pathogen bacteria. These therapies may help restore microbial balance, reduce inflammation, and support periodontal tissue healing [83,84].

Overall, the development of novel therapies for periodontal diseases holds promise for improving patient outcomes, reducing the need for invasive surgical interventions, and addressing the growing burden of periodontal disease on global oral health. Continued research and innovation in this field are essential for advancing the standard of care and providing patients with effective, personalized treatment options for managing periodontitis. 

## 3. Results

An initial comprehensive literature search resulted in 920 items, only 170 remained after the screening. After reading the titles and abstracts and deleting the duplicates, only 102 papers remained. We deleted 24 papers that were published before 2013 and 5 papers that included animal trials. Finally, a total of 78 clinical trials were included in this review, and the results are presented in Table 1, Table 2 and Table 3. None of the participants in the included studies reported side effects from the use of these therapies.

Examples of marketed oral care products are presented in Table 4.

## 4. Discussion

Integrating essential oils, scaffolds, lasers, photodynamic therapy, stem cells, anabolic agents, immunomodulators, colloidal silver nanoparticles, or pre-and probiotics as adjunct therapies in periodontitis can offer additional benefits, complementing conventional approaches and potentially reducing the reliance of antibiotics or other pharmaceutical interventions. As dental professionals, we have to find a way to prevent the symptoms of this disease, so that our patients can keep their natural teeth as long as possible. These effects may impact an individual’s self-esteem and confidence, potentially leading to social withdrawal or the avoidance of social situations. In this matter, these products can help, being easy to use and showing potential in maintaining good oral health. 

However, more research is needed to fully understand their mechanisms of action and optimal application methods in periodontal therapy. Despite this, the growing body of evidence supports their exploration as valuable components of holistic periodontal care.

## 5. Conclusions

Periodontitis prevention and therapy require accurate diagnosis, the elimination of causes, and reductions in modifiable risk factors. Novel approaches to treating periodontitis have demonstrated efficacy and safety as adjunct products, with notable improvements in bleeding on probing and clinical attachment levels attributed to their anti-inflammatory properties. 

The encouraging results from this review support the viability of incorporating these novel therapies, including essential oils, into treatment strategies. Importantly, no short- or long-term side effects have been reported, indicating their safety profiles. However, the lack of awareness regarding periodontal disease’s impact on oral and systemic overall health may hinder treatment compliance, thereby exacerbating the condition’s management. An effective therapeutic approach includes professional intervention, enhanced oral hygiene practices, and ongoing care to mitigate its effect and maintain oral health. Following that, periodontal maintenance therapy and long-term follow-up are crucial to the treatment’s success and tooth retention over time.

Nevertheless, further clinical studies are warranted to elucidate the precise effects of these products on periodontal tissues and determine the optimal utilization methods for preventing periodontal diseases effectively.

## Figures and Tables

**Table 1:** Novel approaches in the therapy of periodontal diseases.

Novel Treatments	Advantages	Disadvantages	Therapeutic Properties	Administration	Reference
Stem cell-based tissue engineering	- enhances healing; - has the ability to differentiate into various cell types; - helps reduce inflammation.	- treatment can be complex; - ethical concerns.	- immunomodulatory potential; - periodontium regeneration.	- intrasulcular application.	[71,72,85,86,87,88,89]
Colloidal silver nanoparticles	- can easily penetrate tissues due to their small size; - reduce inflammation and bacterial load; - reduce the risk of antibiotic resistance development.	- risk of tissue discoloration; - bacteria can develop resistance to silver nanoparticles over time.	- antimicrobial effect on periodontal pathogens.	- intrasulcular application.	[90,91,92]
Pre- and Probiotics	- help reduce dental plaque; - decrease the concentration of *Porphyromonas gingivalis* and gingival inflammation; - can be easily integrated into daily oral care routines; - help maintain oral microbiome health.	- the effects do not last long in the host; - efficacy can be influenced by diet or lifestyle.	- improvement of pocket depth, clinical attachment, and alters anaerobic bacteria colonization.	- oral intake, 1 capsule/day.	[84,93,94,95,96,97,98,99,100]
Immunomodulatory therapy	- helps reduce bone loss by controlling osteolytic and inflammatory processes; - establishes an anti-inflammatory microenvironment that is favorable for bone regeneration; - allows a more focused and precise approach.	- not all patients may respond equally well; - variable in availability and usage; - high costs.	- reduces bone loss; - stops the progression of the disease.	- oral intake.	[66,101,102,103]
Anabolic agents (teriparatide, sclerostin antibody)	- target alveolar bone defects; - have significant outcomes in bone formation; - increase attachment levels; - rebalance osteoblast and osteoclast levels.	- some patients might have contraindications or interactions with other medications; - improper dosage can lead to complications.	- increase bone formation.	- oral intake or intrasulcular application.	[104,105,106]
Lasers	- offer bacterial reduction in periodontal pathogens; - increase attachment levels; - target alveolar bone defects; - control inflammation.	- high costs; - generate heat that can cause pulp damage; - limited tissue penetration; - multiple sessions.	- stimulation of living tissues; - root planning and removal of calculus; - coagulation and hemostasis.	- direct activation at a 1 cm distance from the affected area.	[107,108,109,110,111,112,113,114,115]
Photodynamic therapy	- reduces the risk of antibiotic resistance development; - effective antibacterial action; - controls inflammation; - increases attachment levels.	- limited tissue penetration; - temporary discoloration; - high costs; - multiple sessions.	- eliminates infection in specific tissues.	- the liquid photosensitizer is placed directly in the periodontal pocket.	[116,117,118,119,120,121,122]
Scaffolds	- provide structural support; - increase major attachment levels; - enhance tissue healing and regeneration; - are biocompatible.	- high biodegradation rate; - invasive placements; - risk of complications.	- tissue engineering; - biological, physical, and mechanical properties.	- surgical placement, secured with sutures.	[72,82,86,123,124,125,126]

**Table 2:** Essential oils as adjunct therapies in clinical trials.

Essential Oil	Reference	Administration	No. of Participants	Duration	Results
*Peppermint oil*	[127]	mouthwash (oral rinse)	90	30 days	- better effects when used with conventional mechanical instrumentation compared to chlorhexidine.
	[128]	mouthwash (oral rinse)	67	6 weeks	- pain reduction; - anti-inflammatory effect compared to chlorhexidine or placebo mouthwashes; - 47.1% improvement.
*Chamomile oil*	[129]	oral gel (intra-sulcular application)	30	4 weeks	- significant bleeding reduction compared to chlorhexidine and a placebo product; - reduced plaque accumulation by 39.9%.
*Eucalyptus oil*	[130]	mouthwash (oral rinse)	42	5 months	- chlorhexidine in association with conventional mechanical therapy was more effective than EO monotherapy.
	[131]	mouthwash (oral rinse)	90	30 days	- equal improvements in pocket probing depth, clinical attachment level, gingival index, and bleeding on probing in stages II and III of periodontitis compared to chlorhexidine.
*Tea tree oil*	[132]	mouthwash (oral rinse)	30	14 days	- significant improvements in pocket probing depth, clinical attachment level, gingival index, and bleeding on probing in stage II of periodontitis compared to chlorhexidine.
	[133]	mouthwash	30	20 days	- significant improvements in pocket probing depth, clinical attachment level, gingival index, and bleeding on probing in the early stages of periodontitis compared to chlorhexidine (53%).
*Salvia officinalis oil*	[134]	mouthwash (oral rinse)	338	6 months	- significant inflammation reduction (42.6%) compared to a cetyl-pyridinium chloride-containing mouthwash in the early stages of periodontitis.
*Thyme oil*	[135]	mouthwash (oral rinse)	60	3 months	- effective in pocket probing depth, clinical attachment level, gingival index, and bleeding on probing compared to chlorhexidine and zinc acetate.
*Curcumin oil*	[136]	oral gel (intra-sulcular application)	30	45 days	- has superior efficacy in treating periodontitis (stages I, II, and III) compared to chlorhexidine.
*Rosmarinus officinalis oil*	[137]	mouthwash (oral rinse)	36	8 weeks	- no significant results for gingivitis; - fluoride and chlorhexidine proved to be more effective than EOs on plaque reduction.
*Vetiver grass oil*	[138]	mouthwash (oral rinse)	20	14 days	- proved to be as effective as chlorhexidine regarding probing depth, clinical attachment loss, and gingival bleeding.
*Olive oil and* *St. John’s wort oil*	[139]	mouthwash (oral rinse)	90	6 months	- good results for gingivitis; - EOs showed similar results as chlorhexidine regarding swelling, pain, infectious complications, and periodontal healing.
*Carica papaya leaf extract*	[140]	mouthwash (oral rinse)	138	4 weeks	- good results compared to SLS-free enzyme-containing dentifrice (33%); - reductions in gingival bleeding and inflammation in periodontitis stage I.

**Table 3:** Essential oil oral care products in the therapy of periodontitis.

Oral Product	Essential Oils	Advantages	Disadvantages	Reference
Mouthwashes	*Chamomile oil* *Lemongrass oil*	- reduce gingival bleeding;	- limited tissue penetration;	
	*Zingiber officinalis oil* *Citrus oil* *Zataria multiflora oil* *Peppermint oil* *Curcumin oil* *Calendula officinalis oil*	- help maintain oral microbiome health; - reduce halitosis; - reduce fungal load.	- limited effects on *Candida albicans.*	[140,141,142,143,144,145,146,147,148,149,150,151,152]
Sprays	*Thyme oil* *Ginger oil* *Citrus oil* *Salvia officinalis oil* *Rosmarinus officinalis oil* *Peppermint oil*	- reduce antiviral load; - antifungal properties; - improved gingival bleeding; - increase attachment levels.	- limited tissue penetration; - no traditional use in the oral cavity; - bad taste.	[153,154]
Toothpastes	*Lavender oil*	- can be easily integrated into daily oral care routine; - good taste.	- limited tissue penetration.	
	*Thyme oil*	- reduces plaque adhesion.	- limited antiviral effects.	[140,155,156]
	*Coconut oil*	- reduces inflammation; - increases attachment levels; - reduces bleeding on probing;	- no antitumor effect.	
	*Eucalyptus oil* *Tea tree oil* *Cinnamon oil*	- reduce bacterial load.		
Gels	*Menthol* *Oregano oil* *Lavender oil* *Peppermint oil*	- reduce inflammation and bacterial load; - healing potential; - analgesic potential.	- limited tissue penetration; - multiple sessions.	
	*Thyme oil* *Clove oil* *Cinnamon oil* *Tea tree oil* *Thieves oil* *Lemongrass oil* *Citrus oil* *Eucalyptus oil*	- increase attachment levels; - antitumor effects; - reduce probing depth; - antibacterial and antifungal properties; - significant bacterial inhibition; - significant inhibition of dental plaque formation.	- no effect on *Escherichia coli*; - bad taste.	[78,157,158,159,160,161,162]

**Table 4:** Examples of marketed essential oil oral care products in the therapy of periodontitis in Europe [163].

Product Name	Essential Oils	Topical Pharmaceutical Form	Administration	Indications
Dentosept^®^ (PlantExtrakt, Cluj, Romania)	*Peppermint oil* *Salvia officinalis extract* *Eucalyptus oil* *Thyme oil* *Castor oil*	oral spray	- apply on a cotton swab and rub the affected area or spray directly at a distance of 1 cm from the affected area, up to 5 times/day.	- anti-inflammatory; - antibacterial; - disinfectant; - reduces plaque formation; - reduces gingival bleeding; - adjunctive in periodontitis.
Kamistad^®^ (Stada Arzneimittel AG, Bad Vilbel, Germany)	*Chamomile oil* *Cinnamon oil*	oral gel	- apply directly on inflamed gingiva with a finger or a cotton swab and massage gently up to 3 times/day.	- anti-inflammatory; - reduces gingival bleeding; - antiviral; - antibacterial - reduce halitosis.
Mucosit^®^ (Herbapol S.A., Poznan, Poland)	*Salvia officinalis oil* *Chamomile oil* *Thyme oil*	oral gel	- apply directly on inflamed gingiva with a finger or a cotton swab and massage gently, up to 3 times/day.	- local anesthetic; - accelerates wound healing; - anti-inflammatory; - anti-microbial.
Septosan^®^ (Herbapol S.A., Poznan, Poland)	*Calendula officinalis oil* *Thyme oil* *Chamomile oil*	sachets infusion	- pour 1 sachet in 100 mL of boiling water, infuse for 15 min; mouthwash only, up to 1–5 times/day.	- disinfectant; - anti-inflammatory; - antiviral.
Aperisan^®^ (Dentinox Lenk & Schuppan KG, Berlin, Germany)	*Salvia officinalis oil*	oral gel	- apply directly on inflamed gingiva with a finger or a cotton swab and massage gently, up to 3 times/day.	- anti-inflammatory; - antibacterial.

## Data Availability

Information provided in this research are supported by the inserted references or have been generated by using the softs mentioned in the main text.
